# Priming Neural Circuits to Modulate Spinal Reflex Excitability

**DOI:** 10.3389/fneur.2017.00017

**Published:** 2017-02-03

**Authors:** Stephen P. Estes, Jennifer A. Iddings, Edelle C. Field-Fote

**Affiliations:** ^1^Shepherd Center, Crawford Research Institute, Atlanta, GA, USA; ^2^School of Medicine, Emory University, Division of Physical Therapy, Atlanta, GA, USA

**Keywords:** spasticity, spinal cord injury, stretching, cyclic passive movement, transcutaneous spinal cord stimulation, tanscranial direct current stimulation

## Abstract

While priming is most often thought of as a strategy for modulating neural excitability to facilitate voluntary motor control, priming stimulation can also be utilized to target spinal reflex excitability. In this application, priming can be used to modulate the involuntary motor output that often follows central nervous system injury. Individuals with spinal cord injury (SCI) often experience spasticity, for which antispasmodic medications are the most common treatment. Physical therapeutic/electroceutic interventions offer an alternative treatment for spasticity, without the deleterious side effects that can accompany pharmacological interventions. While studies of physical therapeutic/electroceutic interventions have been published, a systematic comparison of these approaches has not been performed. The purpose of this study was to compare four non-pharmacological interventions to a sham-control intervention to assess their efficacy for spasticity reduction. Participants were individuals (*n* = 10) with chronic SCI (≥1 year) who exhibited stretch-induced quadriceps spasticity. Spasticity was quantified using the pendulum test before and at two time points after (immediate, 45 min delayed) each of four different physical therapeutic/electroceutic interventions, plus a sham-control intervention. Interventions included stretching, cyclic passive movement (CPM), transcutaneous spinal cord stimulation (tcSCS), and transcranial direct current stimulation (tDCS). The sham-control intervention consisted of a brief ramp-up and ramp-down of knee and ankle stimulation while reclined with legs extended. The order of interventions was randomized, and each was tested on a separate day with at least 48 h between sessions. Compared to the sham-control intervention, stretching, CPM, and tcSCS were associated with a significantly greater reduction in spasticity immediately after treatment. While the immediate effect was largest for stretching, the reduction persisted for 45 min only for the CPM and tcSCS interventions. tDCS had no immediate or delayed effects on spasticity when compared to sham-control. Interestingly, the sham-control intervention was associated with significant within-session increases in spasticity, indicating that spasticity increases with immobility. These findings suggest that stretching, CPM, and tcSCS are viable non-pharmacological alternatives for reducing spasticity, and that CPM and tcSCS have prolonged effects. Given that the observed effects were from a single-session intervention, future studies should determine the most efficacious dosing and timing strategies.

## Introduction

In recent years, priming via physical therapeutic and/or electroceutic techniques has been used for modulating neural excitability and improving motor learning (1). While priming is most typically regarded as a strategy to facilitate voluntary motor control in the context of rehabilitation, the use of priming stimulation for the modulation of involuntary motor activity following central nervous system (CNS) injury has received less attention. Priming could likewise be used to target the spinal circuitry, thus modulating the excitability of the spinal reflexes. This type of priming stimulation could be a useful tool for the modulation of aberrant involuntary motor output following CNS injury.

Following spinal cord injury (SCI), disruption in communication between the brain and spinal cord causes an imbalance in the modulatory inputs to spinal reflex circuitry. The consequence of which includes spastic hypertonia and increased reflex excitability, commonly referred to as spasticity, that can impair motor function and diminish quality of life. Antispasmodic medication is the standard treatment for spasticity. While pharmacological treatments can effectively decrease hyperexcitability in the spinal circuitry and normalize exaggerated muscle tone [for review, see Ref. (2)], they are costly and are associated with a number of deleterious side effects, including weakness, lethargy, and drowsiness (3–5), which may limit functional improvements.

Physical therapeutic/electroceutic interventions are non-pharmacological approaches that utilize mechanical and electrical stimulation to modulate the state of excitability of the nervous system (2, 6–10). Importantly, these interventions are often inexpensive and not associated with the negative side effects commonly observed with antispasmodic medications. In the context of spasticity, physical therapeutic/electroceutic interventions can modulate spinal cord inputs and rebalance modulatory influences on the spinal circuitry. Therefore, the priming stimulation provided by these interventions has the potential to modulate involuntary motor output and thereby reduce spasticity.

Stretching is a commonly used physical therapeutic intervention for the management of spasticity (11) that provides constant afferent activation throughout the duration of the stretch. Although the mechanisms by which stretching modulates the spinal reflex circuitry are not well characterized, stretching has been shown to improve clinical measures of spasticity (12) and decrease ankle resistance torque (13) in individuals with spasticity. In contrast to the continuous afferent activation provided during stretching, cyclic passive movement (CPM) primes the spinal circuitry by supplying repeated, patterned afferent input to the spinal cord. The activation of afferent fibers that occurs during movement is thought to play a critical role in spinal reflex modulation, as experimental evidence shows that prolonged joint immobilization causes reflex hyperexcitability in non-injured individuals (14). Moreover, CPM has been shown to improve Modified Ashworth scores (a clinical measure of spasticity) and restore post-activation depression in persons with SCI (8, 15).

While stretching and CPM activate discrete sets of afferent inputs with a temporal pattern that can be varied, transcutaneous spinal cord stimulation (tcSCS) continuously stimulates spinal afferents at multiple segmental levels concurrently. Early work with spinal cord stimulation used implanted epidural electrodes to reduce spasticity in persons with multiple sclerosis (16, 17), stroke (18), and SCI (19, 20). More recently, epidural stimulation of the lumbar spinal cord at a frequency ranging from 50 to 100 Hz has been shown to reduce lower extremity spasticity (21). Stimulation over the spinal cord using tcSCS at 50 Hz has also been shown to reduce lower extremity spasticity in a case series of persons with chronic, incomplete SCI (9). While epidural lumbar spinal cord stimulation and tcSCS both stimulate large diameter dorsal root fibers for the modulation of spinal reflex circuitry (6, 22), tcSCS is a non-invasive method for stimulating the spinal cord circuitry making it a more clinically accessible electroceutic intervention for reducing spasticity.

Because supraspinal inputs contribute to the modulation of spinal reflex excitability, priming stimulation targeting the cortex can be utilized to enhance descending drive to inhibitory circuits in the spinal cord and thus modulate the excitability of the spinal reflex circuitry. Supraspinal circuits have been shown to activate inhibitory mechanisms in the spinal circuitry (23–26). Therefore, non-invasive brain stimulation (NIBS) techniques can be used to target inhibitory spinal circuitry indirectly by increasing the excitability of supraspinal inputs that activate inhibitory spinal circuits. Transcranial direct current stimulation (tDCS) is a form of NIBS that has been shown to increase corticospinal excitability in persons with SCI (27). A recent study demonstrated that excitatory stimulation of the motor cortex with anodal tDCS reduces spasticity in individuals with cerebral palsy (28). To the best of our knowledge, tDCS has not been evaluated as a treatment for spasticity in persons with SCI. However, another form of NIBS, repetitive transcranial magnetic stimulation, has been shown to decrease spasticity in individuals with SCI (10, 29), suggesting that tDCS could potentially improve spasticity post-SCI as well.

While numerous physical therapeutic/electroceutic interventions have been investigated as treatments for spasticity, a systematic comparison of multiple therapies in the same individuals has not been performed. Consequently, the purpose of this study was to compare stretching, CPM, tcSCS, and tDCS with a sham-control to determine their efficacy as physical therapeutic/electroceutic interventions for the reduction of lower extremity spasticity in persons with chronic SCI (>1 year post-injury). Our goal was to determine whether one of these physical therapeutic/electroceutic interventions emerged as a more efficacious therapy for the management of spasticity that could then serve as the basis for continued research.

## Materials and Methods

This study was carried out with approval of the Shepherd Center Research Review Committee. All the participants gave written informed consent prior to study enrollment in accordance with the Declaration of Helsinki.

### Subjects

Individuals were eligible to participate if they met the following inclusion criteria: 18–65 years of age with a chronic SCI (≥1 year post-injury), any International Standards for Neurological Classification of SCI (ISNCSCI) classification level, and self-reported spasticity in the lower extremities. Individuals with the following exclusion criteria were excluded from participation: neurological level of injury below T12, orthopedic problems that would prevent participation in study interventions (i.e., contracture or heterotopic ossification that limited knee or hip range of motion >10°), implanted stimulators of any type, or active infection. Individuals taking prescription medications for management of spasticity were eligible for participation in the study provided that the dosage was stable (no change <2 weeks prior to enrollment).

Of the 18 participants recruited for this study, 10 subjects completed the study with one subject declining to participate in the tDCS session. Participant demographics can be found in Table 1. All participants had SCI of traumatic origin.

**Table 1:** **Participant demographics**.

Participant	Gender	Age (years)	Time since injury	AIS	Neurological injury level	LE tested	Antispastic agents
1	M	29	2 years, 5 months	B	C6	R	Baclofen
2	M	41	6 years, 8 months	C	T6	L	Baclofen
3	M	57	2 years, 9 months	D	C5	L	Baclofen
4	M	45	12 years, 1 month	B	T10	L	None
5	M	49	9 years, 2 months	D	C4	R	Baclofen
6	M	60	2 years, 0 months	D	C4	R	None
7	F	36	2 years, 5 months	D	C6	R	None
8	M	24	4 years, 0 months	C	T7	L	Baclofen
9	M	61	2 years, 8 months	D	C4	L	Baclofen
10	F	60	10 years, 10 months	B	T12	L	None

*AIS, American Spinal Injury Association Impairment Scale; LE, lower extremity*.

### Interventions

We used a randomized crossover design consisting of a single session each of four different physical therapeutic/electroceutic interventions and a sham-control intervention. Sessions were separated by a minimum of 48 h to prevent carryover effects between stimulation types.

#### Stretching

Study physical therapists performed a series of stretches targeting the hip flexors/extensors, knee flexors/extensors, and ankle plantarflexors. Participants were positioned in supine for stretching of the hip extensors, knee flexors, knee extensors, and ankle plantarflexors and repositioned in sidelying with the pelvis in neutral alignment for stretching of the hip flexors. Each stretch position was held for a total of 60 s. Muscles in both lower extremities (i.e., the test and non-test leg) were stretched three times each.

#### Cyclic Passive Movement

A treadmill-mounted robotic gait orthosis (Lokomat, Hocoma Inc., Norwell, MA, USA) was used for CPM of the lower extremities. Participants were secured into the robotic gait orthosis using a body-weight support harness and instructed to remain relaxed and allow the device to move their legs for 30 min. CPM parameters varied among participants. The lowest amount of body-weight support and quickest step speed that an individual could tolerate while maintaining normal gait kinematics (i.e., adequate toe clearance during the swing phase) were utilized.

#### Transcutaneous Spinal Cord Stimulation

Electrical stimulation was applied over vertebral levels T11/T12 using a portable electrotherapy unit (Empi Continuum, DJO Global, Vista, CA, USA). A small round electrode (5 cm) was placed on the back in the area between T11–T12, and one butterfly electrode (10 cm × 15 cm) was placed over the umbilicus in a montage analogous to a previous study investigating the use of tcSCS as a treatment for spasticity (9). Biphasic, 50 Hz stimulation intensity was slowly increased until paresthesia of the lower legs and feet was achieved (for those individuals with sensation below the level of injury) or to the highest level the subject could tolerate without discomfort (for those individuals without sensation below the level of injury). Additionally, target stimulation intensity was subthreshold for lower extremity muscle activation as verified by the absence of visible twitches in lower extremity muscles. Once this target stimulation intensity was reached, stimulation was delivered for 30 min similar to previous work (9).

#### Transcranial Direct Current Stimulation

Non-invasive brain stimulation was delivered using a constant current stimulator (1 × 1 tDCS device, Soterix Medical Inc., New York, NY, USA). Stimulation was applied for 20 min at an intensity of 2 mA as a recent study suggested that this is the necessary stimulation intensity for modulating corticospinal excitability in persons with SCI (27). Using the 10–20 electroencephalographic system, the anode (35 cm^2^) was placed 1 cm anterior to the vertex (Cz), and the cathode (35 cm^2^) was placed over the inion. This electrode montage has been shown to increase corticospinal excitability to both lower extremities concurrently (30).

#### Sham-Control Stimulation

The sham-control condition was designed to detect any non-intervention related study effects (i.e., resting in semi-reclined position with legs elevated, placebo effects). Subjects were seated in a semi-reclined position on a padded, adjustable height mat table with their lower legs extended and resting on a chair. This position was selected as subjects were also seated in the same manner for the other interventions that did not involve movement (i.e., tcSCS and tDCS). Electrodes were placed over the medial and lateral malleoli and the medial and lateral tibial plateaus. Stimulation intensity was increased until the subject could feel the stimulation (for those individuals with sensation below the level of injury). After the sensory threshold was reached, the subjects were told that the stimulation would be decreased to a subthreshold level when, in fact, the stimulation was turned off. Although the stimulation was off for the 30-min session, it was important to deliver a brief period of stimulation at the beginning of the session to provide an input that the subjects could perceive as treatment. To limit the potential effect of movement-related afferent input, subjects remained seated with legs extended for 30 min after the stimulation was stopped.

### Assessment of Spasticity

The lower extremity with greater spasticity, as determined by subject self-report, was identified upon study enrollment. All assessments were conducted in the same lower extremity throughout the duration of the study. For each session, assessments were completed at three time points: (1) prior to the start of the intervention (baseline), (2) immediately after the conclusion of the intervention (immediate), and (3) 45 min after the conclusion of the intervention (delayed). To account for intra-individual differences in spasticity between sessions, the immediate and delayed effects for each intervention were compared to the baseline values obtained immediately prior to the intervention.

**Figure 1:**
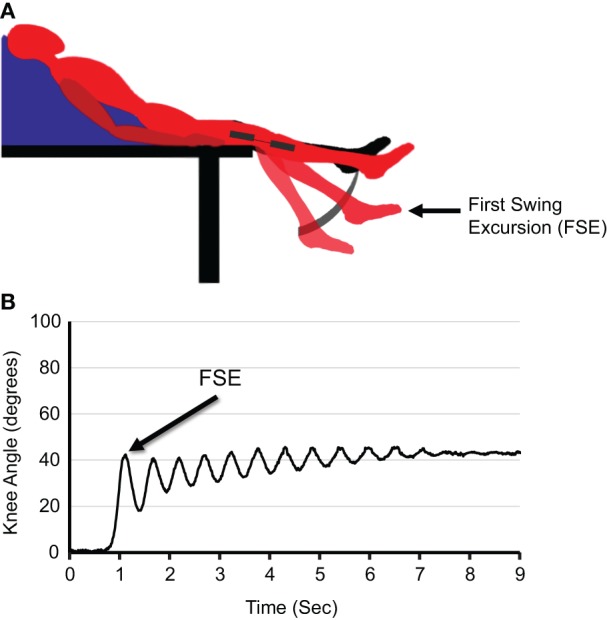
**Quantifying spasticity using the pendulum test**. **(A)** Schematic of pendulum test experimental setup. **(B)** Representative knee angle response profile of an individual with spasticity during the pendulum test. The first swing excursion is defined as the angle at which the lower leg transitions from flexion to extension during after the heel of the test leg is released.

The pendulum test, a biomechanical measure of stretch-induced quadriceps spasticity, which has been shown to correlate with clinical measures of spasticity in persons with SCI (31), was selected as the spasticity measure for this study (see Figure 1A for diagram of experimental setup). Three pendulum test trials were completed at each of the three assessment time points (baseline, immediate, delayed); each trial was separated by a minimum of 30 s. An electrogoniometer (model # SG150, Biometrics Ltd., Newport, UK) strapped to the lateral aspect of the knee joint with neoprene wraps was used to measure joint kinematics, and an electromyographic (EMG) recording electrode (Motion Lab Systems, Baton Rogue, LA, USA) placed over the rectus femoris was used to confirm stretch-induced quadriceps activation during the pendulum test. Spike software (Cambridge Electronic Design Limited, Cambridge, England) was used for acquisition and analysis of joint angle and quadricep EMG data.

The pendulum test was performed as previously described (32). Briefly, the subject was seated on a padded, adjustable height mat table in a semi-reclined position with their lower legs hanging over the edge of the mat. The examiner grasped the participant’s heel and fully extended the test leg. The examiner then released the heel allowing the lower leg to swing. This gravity-induced movement of the lower leg stretches the quadriceps muscle and elicits a stretch reflex-induced contraction of the quadriceps, which is reflected in the knee angle.

The primary outcome measure for this pilot study was the first swing excursion (FSE) of the knee joint during the pendulum test (see Figure 1B for an illustration of the FSE during a representative knee angle response profile). Previous research suggests that FSE is a better indicator of spasticity than other pendulum test components such as the number of oscillations or relaxation index (33). FSE is the angle at which the movement of the lower leg reverses from flexion to extension after release of the heel. This measurement represents the point during gravity-induced knee flexion at which the stretch-induced spasticity of the quadriceps triggers a reflex contraction. An increased FSE represents a decrease in spasticity as the quadriceps stretches to a longer length before a reflex contraction is elicited. For each assessment, the mean of three FSEs was calculated for each participant.

### Data Analysis

SPSS version 22 (IBM, Armonk, NY, USA) was used for all statistical analyses. Data are presented as the mean ± SEM. Significance was set at α = 0.05 for all analyses. Differences between each of the five different interventions were evaluated using repeated measures one-way ANOVA with *post hoc* Bonferroni correction for multiple comparisons. The within-session effects of each intervention were evaluated using Paired Student’s *t*-tests. The effect size of each intervention was calculated using Cohen’s *d*.

## Results

### Treatment Effects Compared to Sham-Control Intervention

#### Immediate Effects

**Figure 2:**
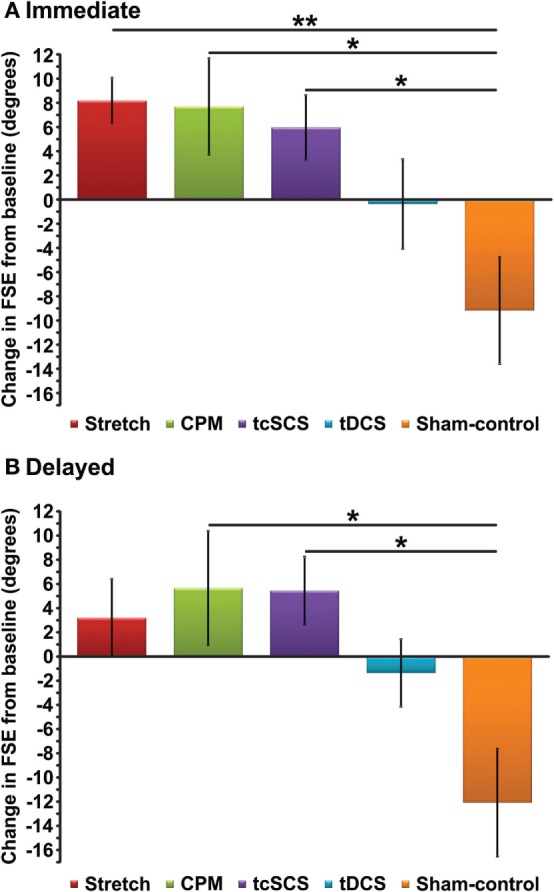
**Change in group mean first swing excursion (FSE) values from baseline to immediate post-test (A) and delayed post-test (B)**. Results represent means ± SEM. *p < 0.05; **p < 0.01.

To determine the immediate effect of each physical therapeutic/electroceutic intervention on spasticity, we first calculated the change in FSE from baseline to immediately posttreatment for each intervention (Figure 2A). When comparing changes in FSE for each intervention to the sham-control intervention at the immediate post-test, we found that a single session of stretching, CPM and tcSCS was associated with a significant mean change in FSE compared to sham-control (Table 2; one-way ANOVA, Bonferroni *post hoc* test, *p* < 0.05). These changes met the criteria for large effect sizes (34) at 1.62 (stretching), 1.27 (CPM), and 1.31 (tcSCS). A single session of tDCS did not result in a significant change in FSE compared to sham-control (Table 2; one-way ANOVA, Bonferroni *post hoc* test, *p* = 0.826).

**Table 2:** **Comparison of change in first swing excursion (FSE) between each physical therapeutic/electroceutic intervention and sham-control**.

	Change in FSE from baseline compared to sham-control	
	Immediate	Delayed
Stretching (*n* = 10)	17.36* (1.62)	15.28 (1.25)
Cyclic passive movement (*n* = 10)	16.87* (1.27)	17.74* (1.23)
Transcutaneous spinal cord stimulation (*n* = 10)	15.15* (1.31)	17.53* (1.49)
Transcranial direct current stimulation (*n* = 9)	8.81 (0.70)	10.72 (0.93)

*Effect size for change in FSE listed in parentheses. Results represent the difference of the mean change in FSE from baseline between each physical therapeutic/electroceutic intervention and the sham-control*.

*p < 0.05.

#### Delayed Effects

To determine the delayed effect of each physical therapeutic/electroceutic intervention on spasticity, we calculated the change in FSE from baseline to 45 min posttreatment (delayed) for each intervention (Figure 2B). In comparing changes in FSE for each intervention to the sham-control intervention at the delayed post-test, we found that only CPM and tcSCS had significant increases in mean FSE compared to sham-control (Table 2; one-way ANOVA, *p* < 0.05) with effect sizes of 1.23 (CPM) and 1.49 (tcSCS). While stretching showed immediate changes in FSE, these effects appeared to be short term, as the change in FSE showed no significant difference compared to sham-control 45 min after treatment (Table 2; one-way ANOVA, Bonferroni *post hoc* test, *p* = 0.052). It should be noted, however, that stretching had an effect size of 1.25, indicating a large effect. tDCS was found to have no significant delayed effect on the FSE compared to sham-control (Table 2; one-way ANOVA, Bonferroni *post hoc* test, *p* = 0.510).

There was no significant difference in FSE when comparing each physical therapeutic/electroceutic intervention to each other at either the immediate or delayed post-test (one-way ANOVA, Bonferroni *post hoc* test, *p* > 0.10), indicating that no one physical therapeutic/electroceutic treatment provided greater reduction in spasticity than another.

### Treatment Effects within Intervention

**Figure 3:**
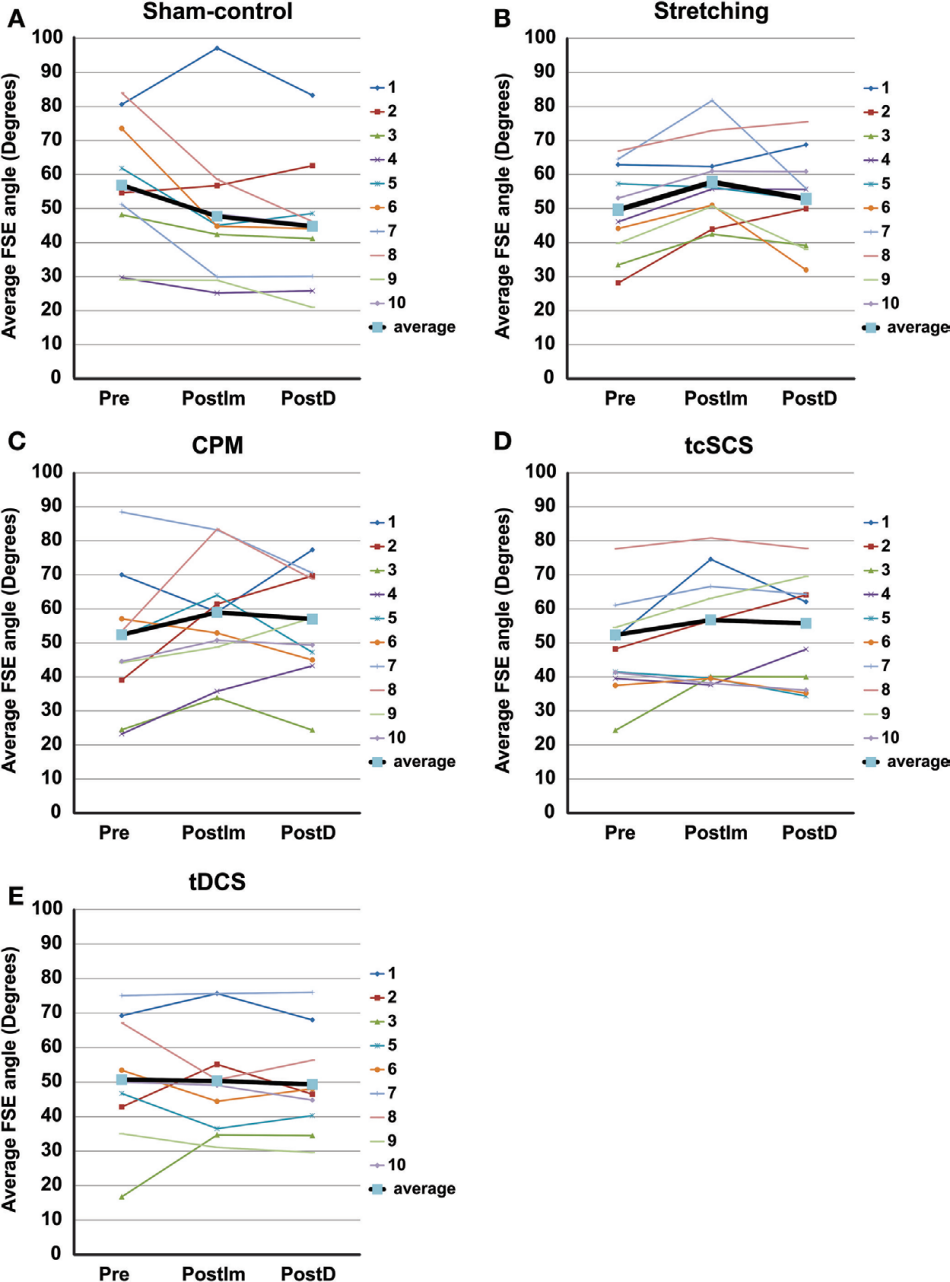
**Within-session first swing excursion (FSE) values for each intervention: (A) sham-control, (B) stretching, (C) cyclic passive movement (CPM), (D) transcutaneous spinal cord stimulation (tcSCS), and (E) transcranial direct current stimulation (tDCS)**. In each panel, the thin, colored lines display individual results for each participant and the bold black line displays the mean for all participants. One participant declined to participate in tDCS; hence, there are only nine participants displayed for this intervention. Individual results represent means of three pendulum tests and group means represent the mean of all individual trials. Abbreviations: Pre, baseline; PostIm, immediate post-test; PostD, delayed post-test. *p < 0.05; **p < 0.01.

In comparing changes in FSE within each intervention, we found that the mean FSE decreased following the 30-min sham-control intervention (Table 3; Figure 3A), indicating an increase in spasticity. While a majority of subjects showed a decrease in FSE after sham-control treatment, two subjects had an increase in FSE suggestive of a placebo effect of the sham-control intervention for these individuals. Further analysis of the within-session effects of the sham-control intervention showed that there was a significant decrease in the mean FSE from baseline to 45 min after treatment (paired-*t*-test, *t* = 2.718, *p* = 0.024) with a moderate effect size of 0.65. Taken together, these findings indicate that inactivity, as occurred in the sham-control, may worsen spasticity.

**Table 3:** **Group mean first swing excursion (FSE) values for each intervention**.

	FSE		
	Baseline	Immediate	Delayed
Stretching (*n* = 10)	49.62 ± 4.26	57.80 ± 3.89** (0.63)	52.83 ± 4.34 (0.24)
Cyclic passive movement (*n* = 10)	49.63 ± 6.23	57.32 ± 5.34 (0.42)	55.29 ± 5.19 (0.31)
Transcutaneous spinal cord stimulation (*n* = 10)	47.70 ± 4.64	53.67 ± 5.29 (0.38)	53.16 ± 5.12 (0.35)
Transcranial direct current stimulation (*n* = 9)	50.70 ± 6.12	50.33 ± 5.46 (0.02)	49.34 ± 5.04 (0.08)
Sham-control (*n* = 10)	56.88 ± 5.99	47.70 ± 6.55 (0.46)	44.80 ± 5.75* (0.65)

Effect size for within-session pre- and post-test comparisons listed in parentheses. Results represent means ± SEM. *p < 0.05, **p < 0.01.

Within-treatment assessments of each physical therapeutic/electroceutic intervention showed that the effects of each treatment varied across subjects (Figure 3). Analysis of the within-treatment effects of a single stretching session showed that the FSE increased in 9/10 subjects (Figure 3B). Of those responders, two showed an increase in FSE of 12 ° or greater, which has been identified as a clinically meaningful difference in FSE as observed in a study of the antispasmodic tizanidine (31). Six subjects showed a moderate increase in FSE of 6–11°, and one showed a mild increase in FSE of 1–5°. On average, stretching resulted in a statistically significant increase in FSE from baseline to immediately after stretching (Table 3; paired-*t*-test, *t* = −4.354, *p* = 0.002) with a moderate effect size. There was no significant difference in FSE at the delayed post-test (Table 3; paired *t*-test, *t* = −1.005, *p* = 0.341), indicating the effects were short term.

Analysis of a single 30-min CPM session showed that the mean FSE increased in 8/10 subjects (Figure 3C). Of those eight responders, five showed a clinically meaningful increase in FSE of 12° or greater and three showed a moderate increase in FSE of 6–11°. Even though a number of subjects showed individual increases in their FSE after CPM for either the immediate or delayed post-tests, there was no significant mean increase in FSE from baseline at the immediate (Table 3; paired *t*-test, *t* = −1.932, *p* = 0.085) or delayed post-tests (Table 3; paired *t*-test, *t* = −1.202, *p* = 0.260).

Results were similar following 30 minutes of tcSCS in which 8/10 subjects showed an increase in FSE (Figure 3D). Of the eight responders, four individuals had a clinically meaningful increase in FSE of 12° or greater, one individual had a moderate increase in FSE of 6–11°, and three individuals showed the least benefit with an increase in FSE of 1–5°. Analysis of the mean FSE did not show a statistically significant increase in FSE from baseline to immediately after tcSCS (Table 3; paired *t*-test, *t* = −2.249, *p* = 0.051) nor from baseline to 45 min after tcSCS treatment (Table 3; paired *t*-test, *t* = −1.946, *p* = 0.083). While the within-treatment effects of tcSCS were not statistically significant, it should be noted that 4/10 subjects reported feeling a general reduction in spasticity and stiffness following tcSCS that persisted into the evening. No other intervention was reported to have these extended effects.

Stimulation over the motor cortex with tDCS for 20 min increased the FSE in 3/9 subjects, with two subjects showing a clinically meaningful increase in FSE and one subject showing a moderate increase in FSE (Figure 3E). When assessing the mean FSE, we found that there was no significant difference in FSE from baseline to either posttreatment time point (Table 3; immediate: paired-*t*-test, *t* = 0.099, *p* = 0.923; delayed: paired-*t*-test, *t* = 0.488, *p* = 0.639).

In addition to the inter-individual variability in intervention responses described in the preceding paragraphs, we also observed intra-individual variability in baseline FSE from session to session (Table 3; Figure 3). However, baseline FSE values were not different between any of the interventions, including the sham-control (one-way ANOVA, *p* = 0.795).

## Discussion

The purpose of the current study was to compare the efficacy of four physical therapeutic/electroceutic interventions for priming the spinal reflex circuitry to reduce spasticity. Our results showed that physical therapeutic/electroceutic interventions can modulate the spinal reflex circuitry to reduce quadriceps spasticity, as measured by an increase in the knee angle at which the quadriceps muscles first elicit a reflex muscle contraction (i.e., the FSE). When compared to the sham-control therapy, we observed a significant change in FSE (i.e., a decrease in spasticity) following stretching, CPM, and tcSCS at the immediate post-intervention assessment. tDCS showed no significant change in FSE as compared to sham-control. Furthermore, only CPM and tcSCS showed a persistent change in FSE 45-min post-intervention when compared to the sham-control. Secondary analyses of the within-treatment effects for each intervention showed that the sham-control intervention resulted in a significant decrease in FSE, indicating that spasticity worsens when an individual sits for an extended period of time. Taken together, these results suggest that single physical therapeutic/electroceutic interventions, including stretching, CPM and tcSCS, can be used to temporarily prime the spinal cord circuit and modulate reflex excitability for decreased spasticity.

While the specific neurophysiological mechanisms underlying spasticity are disputed, it is generally accepted that spasticity arises from an imbalance in the excitatory and inhibitory inputs into the spinal cord circuitry and that modifying the activity of these inputs can rebalance the circuit (2). Of the interventions tested in the current study, only those that involved the activation of sensory afferents (stretching, CPM, and tcSCS) reduced spasticity. Others have also shown afferent stimulation *via* stretching (12), CPM (8, 15), and tcSCS (9) decreases spasticity. Given evidence that electrical stimulation of sensory afferents remodels the excitability of the spinal reflex circuitry by both direct (spinal level) and indirect (cortical level) mechanisms (19, 21, 35), it may be possible that the direct modulation of sensory afferents results in larger changes in FSE than approaches that act *via* indirect afferent modulation, such as tDCS (36).

Interestingly, the value of afferent stimulation does not appear to be dependent on the type of afferent input used. Even though each intervention activates different populations of sensory afferents in a different fashion (i.e., stretching continually activates muscle afferents, CPM continually activates muscle afferents in a pattern, and tcSCS repeatedly activates afferents across multiple spinal segments), we observed no significant differences in the change of FSE between stretching, CPM, or tcSCS, indicating that these interventions have similar efficacy as primers of spinal cord excitability. The value of these physical therapeutic/electroceutic interventions may, therefore, lie in their individual accessibility, cost, and duration. For most persons with spasticity, stretching is likely to be the most accessible and low cost option. However, our finding that stretching only significantly increased FSE immediately following treatment indicates that the duration of these effects are limited, which may be a limitation for spasticity management if continual stretching is required. With an increase in FSE persisting for 45 min posttreatment, CPM provides a more long-term reduction in spasticity than stretching. However, CPM requires costly equipment and is not readily portable. The increase in FSE induced by tcSCS also persisted for 45 min posttreatment, indicating the potential for more long-term treatment effects. Further, the electrotherapy units used to deliver tcSCS are both more affordable and more portable than CPM devices making them more accessible. This rationale suggests that tcSCS may be the most valuable form of afferent stimulation for the reduction of stretch-induced spasticity, as measured solely by a change in FSE.

Using priming stimulation to target the supraspinal circuitry directly, we did not observe a significant change in FSE when comparing tDCS to sham-control. As highlighted above, afferent stimulation provides dual activation of the spinal circuitry, both direct and indirect, whereas tDCS theoretically activates inhibitory pathways in spinal reflex circuitry indirectly. While our results suggest that solely targeting supraspinal inputs is less effective than afferent stimulation for reducing stretch-induced quadriceps spasticity, NIBS should not be ruled out as a potential physical therapeutic/electroceutic intervention for spasticity management. Other studies investigating the use of NIBS for spasticity reduction have utilized a minimum of five stimulation sessions (10, 28, 29), indicating that higher doses of NIBS may be necessary to modulate spasticity. In the context of priming voluntary motor control, tDCS has been shown to be more efficacious when applied concurrently with task practice than when applied prior to task practice (37–40). Therefore, it is possible that tDCS may be a more efficacious priming stimulation for the modulation of involuntary motor activity when used in combination with another physical therapeutic/electroceutic intervention such as those that target afferent input.

Cortical topography is also a potential mediator of the efficacy of tDCS as a treatment for spasticity. To the best of our knowledge, anodal tDCS has been investigated as a physical therapeutic/electroceutic intervention for the management only of upper extremity spasticity (28). In the current study, we targeted the leg representation of the motor cortex by applying stimulation over the vertex. However, the depth of current penetration with tDCS may make it more difficult to target the leg representation of the motor cortex than the arm and hand representation (41). The orientation and position of different limb representations in the motor cortex may potentially explain why we did not observe an effect of anodal tDCS on lower extremity spasticity when this type of priming stimulation has been shown to have an effect on upper extremity spasticity (28).

Interestingly, we observed a significant decrease in FSE (i.e., increase in spasticity) from baseline to 45 min post-intervention during the sham-control intervention when analyzing within-treatment effects. This reduction in FSE while relaxing in a semi-reclined position with the legs extended suggests that immobility increases stretch-induced quadriceps spasticity. In support of this idea, prolonged joint immobilization has been shown to induce spinal reflex hyperexcitability in non-injured individuals (14). Anecdotally, it is widely acknowledged in clinical circles that spasticity is worst in the morning after individuals have been in the same position for several hours overnight. Because the afferent activation that occurs during movement plays an important role in the modulation of spinal reflex excitability (42), immobility likely increases spasticity due to a lack of afferent input. Our results suggest that individuals with spasticity should be encouraged to be as active as possible.

Given that this was an exploratory study, our conclusions are limited by the small number of participants, and additional studies are needed to corroborate our findings. It should be emphasized that each physical therapeutic/electroceutic intervention was applied only for a single session. While this precludes our ability to make direct comparisons with long-term treatments for spasticity, our short-term results are promising in that we observed significant reductions in spasticity following single sessions of three physical therapeutic/electroceutic interventions, stretching, CPM and tcSCS. It should also be noted that while we applied tcSCS in a manner that would be more clinically accessible, i.e., without the verification of electrode placement using a posterior root motor reflex as conducted in previous studies utilizing tcSCS (9), it may have been possible to enhance the observed effects of tcSCS by verifying the placement. Furthermore, we did not assess segmental reflexes to determine the integrity of the lower motoneurons. However, the presence of lower extremity spasticity indicates that the motoneurons of interest for this study were intact. We acknowledge that we measured a single manifestation of spasticity (i.e., involuntary, stretch-induced muscle activation) in one muscle group using a single outcome measure. For a more complete picture of the efficacy of these physical therapeutic/electroceutic interventions in the treatment of spasticity, other manifestations of spasticity, beyond stretch-induced spasticity, should be evaluated.

It is important to reiterate the inter-individual differences observed with each physical therapeutic/electroceutic intervention upon comparison to the sham-control. Immediately following treatment, the largest increase in FSE was observed following stretching for 4/10 subjects, following CPM for 4/10 subjects, following tcSCS for 1/10 subjects, and following tDCS for 1/10 subjects. At the delayed assessment, CPM and tcSCS were each the most effective intervention for 4/10 subjects, while stretching and tDCS were each the most effective intervention for 1/10 subjects. When comparing differences in intervention efficacy among similar neurological levels of injury, CPM was the most effective intervention for 3/4 individuals with thoracic injury and stretching was the most effective intervention for the remaining one individual with thoracic injury at both the immediate and delayed post-intervention assessments. For the six individuals with a cervical injury, the most effective interventions at the immediate post-intervention assessment were stretching (3/6), CPM (1/6), tcSCS (1/6), and tDCS (1/6). At the delayed post-intervention assessment, tcSCS was the most effective intervention for 4/6 individuals with a cervical SCI. The remaining 2/6 individuals were divided so that CPM was the most effective intervention for one individual and tDCS was the most effective intervention for the other. These inter-individual differences in responsiveness to different treatments suggest that spasticity cannot be managed using a “one size fits all approach;” the same intervention is not likely to work for everyone. Because the efficacy of a given treatment is likely to be dependent on the clinical manifestation(s) of spasticity that an individual is experiencing, future research should be dedicated to characterizing the mechanisms underlying the various clinical manifestations of spasticity.

## Conclusion

In individuals with spasticity, aberrant involuntary motor activity can negatively impact quality of life. Because spasticity can cause stiffness and discomfort and interfere with an individual’s ability to position themselves, it has the potential to have a detrimental effect on a person’s ability to execute residual functional movements. While priming stimulation has previously been investigated as a means to facilitate voluntary motor control, in the current study we showed that it can also be used to modulate involuntary motor activity. We demonstrated that stretching, CPM and tcSCS can be utilized to prime the spinal reflex circuitry and reduce stretch-induced quadriceps spasticity. Our results suggest that physical therapeutic/electroceutic interventions may be able to reduce spasticity to an extent that is comparable to that previously shown for pharmacological treatments. These approaches may therefore provide a means of spasticity management that is both cost-effective and free from the deleterious side effects that accompany many antispasmodic medications. Future research should be dedicated to determining the most efficacious dosing strategies for these physical therapeutic/electroceutic interventions. Moreover, the efficacy of combined physical therapeutic/electroceutic interventions should be evaluated to determine whether applying two types of stimulation concurrently has a synergistic effect.

## Author Contributions

EF-F, SE, and JI designed research. SE and JI performed research and analyzed data. EF-F, SE, and JI wrote the manuscript.

## Conflict of Interest Statement

The authors declare that the research was conducted in the absence of any commercial or financial relationships that could be construed as a potential conflict of interest.
